# From cellular diversity to coordinating nervous system function: the next frontier in non-neuronal cell biology

**DOI:** 10.3389/fncel.2026.1934284

**Published:** 2026-07-28

**Authors:** Jason R. Plemel

**Affiliations:** 1 Neuroscience and Mental Health Institute, University of Alberta, Edmonton, AB, Canada; 2 Multiple Sclerosis Centre, University of Alberta, Edmonton, AB, Canada; 3 Department of Medicine, Division of Neurology, University of Alberta, Edmonton, AB, Canada; 4 Department of Medical Microbiology and Immunology, University of Alberta, Edmonton, AB, Canada; 5 Li Ka Shing Institute of Virology, University of Alberta, Edmonton, AB, Canada

**Keywords:** astrocytes, border cells, glia, microglia, non-neuronal cells, oligodendrocyte, OPC

Over the past decade, non-neuronal cells have emerged from supporting actors to central regulators of nervous system function. Advances in imaging, single-cell genomics, spatial transcriptomics, and genetic manipulation have revealed that non-neuronal cells such as astrocytes, microglia, oligodendrocyte lineage cells, and central nervous system (CNS) border-associated cells actively regulate neural circuit development, plasticity, repair, and disease progression. In the CNS, while neurons transmit information, non-neuronal cells regulate neuronal homeostasis, disease, and even neuronal excitability. These non-neuronal cells enable action potentials at greater speeds, modulate synapses during development and disease, provide metabolic and nutrient support to neurons, and regulate neuronal activity, to name just a few of their roles. In disease states, such as neurodegenerative diseases like Alzheimer's disease, most of the risk alleles are expressed by non-neuronal cells, suggesting these cells are critical contributors to neurodegeneration. With the growing realization of the critical role for non-neuronal cells, this research area is expanding into exciting horizons.

A defining challenge in neuroscience is understanding how non-neuronal cells detect neuronal activity and integrate this information. Understanding how different glial populations integrate neuronal activity into coordinated tissue-level responses may represent one of the central questions in systems neurobiology. For example, astrocytes are situated at synapses, potentially providing contextual integration. Termed contextual guidance, astrocytes may act as “contextual gates” that shape neuronal function and circuitry (Murphy-Royal et al., 2023). The molecular mechanism of how astrocytes integrate information to regulate neurons and whether this contributes to behavior or cognition is an exciting area of research. Microglia, the brain's surveyors, also respond to neuronal information, responding to both more and less neuronal activity (Liu et al., 2019; Umpierre et al., 2020). More still, OPCs form synapses with neurons, directly monitoring neuronal activity (Bergles et al., 2000). Given their proximity to axons through their overlying myelin sheath, oligodendrocytes can also respond to activity along axons (Micu et al., 2018). With each of these cells monitoring neuronal activity, how is this information integrated and how does it impact the response of these non-neuronal cells? Astrocytes are postulated to integrate contextual information, yet it remains unclear how many signals are integrated independently and how information is shared among non-neuronal cells. Defining the non-neuronal cell integratome is critical to understanding CNS function.

Non-neuronal cells do not simply detect neuronal activity; they can regulate neuronal plasticity and behavior. One way these cells regulate neuronal plasticity is by removing synapses. For example, microglia clear synapses during development and disease through complement-dependent and independent mechanisms (Pereira-Iglesias et al., 2025). These processes are regulated by neuronal activity. Microglial synaptic clearance has been understood in several circuits, but how this synaptic clearance relates more broadly to brain and spinal cord plasticity, cognition and behavior is still being defined. Astrocytes and oligodendrocyte progenitor cells (OPCs) are also involved in synapse removal (Chung et al., 2013; Lee et al., 2021; Auguste et al., 2022), yet less is known about how the coordination of synapses occurs between glial cell types. In addition to synaptic restructuring, non-neuronal cells can change neuronal plasticity by other mechanisms. Microglia regulate the breakdown of ATP to adenosine to modulate neuronal activity (Badimon et al., 2020). Non-neuronal cells also produce and maintain perineuronal nets (Tewari et al., 2022), impacting neuronal plasticity (Tewari et al., 2024). For example, the degradation of perineuronal nets after peripheral nerve injury by microglia promotes spinal nociceptive circuits that drive pain hypersensitivity (Tansley et al., 2022). How microglia regulate perineuronal nets during disease and homeostatic processes, with implications for behavior and cognition, is also an exciting area of research.

Another emerging area of glial-neuronal interaction concerns how oligodendrocyte lineage changes following experience and learning, known as myelin plasticity (Xin and Chan, 2020). Myelin is continually changed throughout lifespan (Hughes et al., 2013; Hill et al., 2018; Hughes et al., 2018) in response to sensory experience or neuronal activity (Gibson et al., 2014; Hughes et al., 2018). Myelin plasticity is critical for different types of learning and memory. For example, the conditional blockade of oligodendrocyte production impairs memory consolidation and motor learning, potentially other neurological functions (McKenzie et al., 2014; Pan et al., 2020; Steadman et al., 2020). However, what oligodendrocyte function accounts for these cognitive roles is still unknown. Potentially myelin alters conduction velocity and circuit synchrony, contributing to these behavioral consequences. Alternatively, other oligodendrocyte roles, such as their metabolic coupling to axons (Micu et al., 2018), may affect learning, memory, or cognition. Understanding how oligodendrocytes contribute to behavior is important given the link with myelinating cells and to neurodegenerative and psychiatric diseases such as schizophrenia (Gibson et al., 2018; Nave et al., 2023).

Given the critical roles of non-neuronal cells during homeostasis, the loss of these functions in disease or the acquisition of new roles are central to all neurological diseases. For example, microglia constantly extend their processes into their area of influence, responding to hundreds, if not thousands, of factors (Hickman et al., 2013). During disease, the homeostatic signature of microglia and their surveillant roles shift; microglia transition into many different disease-related states (Zia et al., 2020; Healy et al., 2022; Paolicelli et al., 2022). These states represent a complex response that can be initiated by damage-related molecules such as myelin debris, dead cells, or amyloid (Dolan et al., 2022; Zia et al., 2022). By taking up these disease-related roles, microglia shift their functions in conjunction with state changes. Whether these states all have distinct functions is suspected based on the unique expression of different ligands, but the functions of these microglial states remain largely unexplored. One of the defining challenges of the next decade will be moving beyond transcriptomic descriptions of microglial states toward identifying the molecular switches that govern state transitions—also known as microglial plasticity—and determining which states causally influence neurological disease. In chronic diseases, microglia can form foamy cells with lipid-rich inclusions (Van Der Vliet et al., 2026), as in atherosclerotic plaques, which may represent a point of microglial exhaustion (Chen et al., 2025; Ziaee et al., 2026). Another major challenge will be to understand the biological underpinnings of how microglia fatigue, or become exhausted, in chronic conditions, whether these foamy microglia represent an exhausted or a more reversible “full” microglial state, and whether this is relevant to aging and neurodegenerative conditions.

Emerging is the understanding that all non-neuronal cells shift into different states. Disease-related states are being identified, including disease-associated oligodendrocytes (DAO), disease-associated OPCs, and disease-associated astrocytes (Falcao et al., 2018; Jakel et al., 2019; Habib et al., 2020; Absinta et al., 2021; Patani et al., 2023). These disease-associated states are diverse, but likely none more so than in astrocytes, where many reactive astrocytic states vary with pathology (Patani et al., 2023). These disease-associated states often alter gene expression patterns indicative of innate immune activation, cytokine and chemokine secretion, or type I interferon signaling (Falcao et al., 2018; Kirby et al., 2019; Habib et al., 2020; Absinta et al., 2021; Pandey et al., 2022). During disease conditions, non-neuronal cells respond by upregulating inflammatory molecules involved in antigen presentation, such as MHC molecules (Falcao et al., 2018; Kirby et al., 2019; Absinta et al., 2021; Hill et al., 2026). Other common attributes include lipid remodeling and an oxidative stress response (Falcao et al., 2018; Ioannou et al., 2019; Jakel et al., 2019; Kirby et al., 2019; Absinta et al., 2021; Guttenplan et al., 2021; Hasel et al., 2021; Pandey et al., 2022). Importantly, the identity of disease-associated states consistently reflects a loss of homeostatic identity, suggesting that, in addition to gaining immune functions in disease, non-neuronal cells lose homeostatic functions. Future work should define these disease-associated states using orthogonal tools, in diverse disease conditions, to compare commonalities and differences. Disease-associated cells may gain new functions, but it is also critical to understand how loss of homeostatic functions relates to disease pathogenesis. Understanding non-neuronal cells in disease states must also move beyond defining disease-related states and work toward uncovering the triggers that transition non-neuronal cells into these disease-associated states, identifying the functions of these states, and determining which represent adaptive repair programs and which actively drive pathology.

Beyond the major glial populations of the CNS, important advances are also emerging for non-neuronal cells in the peripheral nervous system (PNS), including Schwann cells, satellite glial cells, enteric glia, and peripheral nerve-resident immune cells. These cells regulate axonal maintenance, regeneration, neuroimmune communication, and neuronal excitability, highlighting the broad importance of non-neuronal biology throughout the nervous system. While major glial cells have many known functions, less investigated are the other CNS cells that largely populate CNS boundaries. For example, in the CNS, various cells surround ventricles and line blood vessels. Additional cells are found layering the pia matter and within the arachnoid and meninges. For example, within the choroid plexus, surrounding blood vessels, and lining the PIA are populations of macrophages referred to as border-associated macrophages (BAM) (Goldmann et al., 2016; Ajami et al., 2018; Kierdorf et al., 2019). Several other barrier cell types are found in the CNS, including endothelial cells, pericytes, choroid plexus epithelial cells, arachnoid barrier cells, ependymal cells, and fibroblasts. Together, these cells regulate molecular transport, immune surveillance, tissue homeostasis, and communication between the CNS, the lymphatics, and the circulation. In addition to barrier cells, there is a complex atlas of adaptive and innate immune cells within the leptomeningeal space and dura mater of the CNS (Mrdjen et al., 2018; Rustenhoven and Kipnis, 2022). While the anatomy of these structures and the diverse cellular components are being defined, how these cells contribute during CNS development, homeostasis, and disease is an emerging research area. Future research investigating the different ways in which barrier cells affect CNS functions, immune cell trafficking, debris clearance, and immune cell activity will provide a new understanding and likely novel targets for treating neurological diseases.

Tools are expanding our understanding of non-neuronal cells. The next decade of non-neuronal cell research will move beyond descriptive cellular atlases toward mechanistic understanding of how diverse glial and border-associated cell populations coordinate neural circuit function, repair, and disease progression. The next generation of approaches, including spatial transcriptomics, *in vivo* imaging, perturbation sequencing, and multimodal profiling, will allow investigators to determine how non-neuronal cells communicate, transition between functional states, and causally regulate behavior and disease. New advances, such as single-cell proteomics, will better define how cell states relate to cellular function. Needed now in the field of non-neuronal cells are tools that enable circuitry-specific manipulation of non-neuronal cells. Tools to define how non-neuronal cells impact specific neurons or circuits will usher in a new era of discovery, linking non-neuronal cells to specific CNS functions.

Over the past century, neuroscience has largely been neuron-centric. The coming decade may instead define neuroscience through understanding how diverse non-neuronal cells coordinate nervous system function across health, aging and disease. Historically, astrocytes, microglia, oligodendrocytes and OPCs have largely been investigated independently. Increasingly, however, evidence suggests these cells function as coordinated cellular networks that collectively sense neuronal activity, maintain homeostasis and orchestrate responses to injury. Understanding this intercellular communication may ultimately prove more informative than studying individual cell types in isolation. Neurological conditions represent a breakdown of homeostasis due to injury, infection, or disease. Given the fundamental role of non-neuronal cells in homeostasis, expanding our knowledge of these critical cells will lead to a greater understanding of disease mechanisms and new strategies to treat neurological conditions. With so much passionate research in neuroscience, the research area of non-neuronal cells is bright.

## References

[B1] Absinta M. Maric D. Gharagozloo M. Garton T. Smith M. D. Jin J. . (2021). A lymphocyte–microglia–astrocyte axis in chronic active multiple sclerosis. Nature. 597, 709–714. doi: 10.1038/s41586-021-03892-7 34497421 PMC8719282

[B2] Ajami B. Samusik N. Wieghofer P. Ho P. P. Crotti A. Bjornson Z. . (2018). Single-cell mass cytometry reveals distinct populations of brain myeloid cells in mouse neuroinflammation and neurodegeneration models. Nat. Neurosci. 21, 541–551. doi: 10.1038/s41593-018-0100-x 29507414 PMC8629134

[B3] Auguste Y. S. S. Ferro A. Kahng J. A. Xavier A. M. Dixon J. R. Vrudhula U. . (2022). Oligodendrocyte precursor cells engulf synapses during circuit remodeling in mice. Nat. Neurosci. 25, 1273–1278. doi: 10.1038/s41593-022-01170-x 36171430 PMC9534756

[B4] Badimon A. Strasburger H. J. Ayata P. Chen X. Nair A. Ikegami A. . (2020). Negative feedback control of neuronal activity by microglia. Nature 586, 417–423. doi: 10.1038/s41586-020-2777-8 32999463 PMC7577179

[B5] Bergles D. E. Roberts J. D. Somogyi P. Jahr C. E. (2000). Glutamatergic synapses on oligodendrocyte precursor cells in the hippocampus. Nature 405, 187–191. doi: 10.1038/35012083 10821275

[B6] Chen J. Q. A. Mcnamara N. B. Engelenburg H. J. Jongejan A. Wever D. D. Hopman K. . (2025). Distinct transcriptional changes distinguish efficient and poor remyelination in multiple sclerosis. Brain 148, 2201–2217. doi: 10.1093/brain/awae414 39718981 PMC12129743

[B7] Chung W. S. Clarke L. E. Wang G. X. Stafford B. K. Sher A. Chakraborty C. . (2013). Astrocytes mediate synapse elimination through MEGF10 and MERTK pathways. Nature 504, 394–400. doi: 10.1038/nature12776 24270812 PMC3969024

[B8] Dolan M.-J. Therrien M. Jereb S. Kamath T. Atkeson T. Marsh S. E. . (2022). A resource for generating and manipulating human microglial states in vitro. bioRxiv 2022.2005.2002.490100. doi: 10.1101/2022.05.02.490100

[B9] Falcao A. M. Van Bruggen D. Marques S. Meijer M. Jakel S. Agirre E. . (2018). Disease-specific oligodendrocyte lineage cells arise in multiple sclerosis. Nat. Med. 24, 1837–1844. doi: 10.1038/s41591-018-0236-y 30420755 PMC6544508

[B10] Gibson E. M. Geraghty A. C. Monje M. (2018). Bad wrap: myelin and myelin plasticity in health and disease. Dev. Neurobiol. 78, 123–135. doi: 10.1002/dneu.22541 28986960 PMC5788316

[B11] Gibson E. M. Purger D. Mount C. W. Goldstein A. K. Lin G. L. Wood L. S. . (2014). Neuronal activity promotes oligodendrogenesis and adaptive myelination in the mammalian brain. Science 344:1252304. doi: 10.1126/science.1252304 24727982 PMC4096908

[B12] Goldmann T. Wieghofer P. Jordao M. J. Prutek F. Hagemeyer N. Frenzel K. . (2016). Origin, fate and dynamics of macrophages at central nervous system interfaces. Nat. Immunol. 17, 797–805. doi: 10.1038/ni.3423 27135602 PMC4968048

[B13] Guttenplan K. A. Weigel M. K. Prakash P. Wijewardhane P. R. Hasel P. Rufen-Blanchette U. . (2021). Neurotoxic reactive astrocytes induce cell death via saturated lipids. Nature 599, 102–107. doi: 10.1038/s41586-021-03960-y 34616039 PMC12054010

[B14] Habib N. Mccabe C. Medina S. Varshavsky M. Kitsberg D. Dvir-Szternfeld R. . (2020). Disease-associated astrocytes in Alzheimer's disease and aging. Nat. Neurosci. 23, 701–706. doi: 10.1038/s41593-020-0624-8 32341542 PMC9262034

[B15] Hasel P. Rose I. V. L. Sadick J. S. Kim R. D. Liddelow S. A. (2021). Neuroinflammatory astrocyte subtypes in the mouse brain. Nat. Neurosci. 24, 1475–1487. doi: 10.1038/s41593-021-00905-6 34413515

[B16] Healy L. M. Zia S. Plemel J. R. (2022). Towards a definition of microglia heterogeneity. Commun. Biol. 5:1114. doi: 10.1038/s42003-022-04081-6 36266565 PMC9585025

[B17] Hickman S. E. Kingery N. D. Ohsumi T. K. Borowsky M. L. Wang L. C. Means T. K. . (2013). The microglial sensome revealed by direct RNA sequencing. Nat. Neurosci. 16, 1896–1905. doi: 10.1038/nn.3554 24162652 PMC3840123

[B18] Hill E. J. Sojka C. Sampson M. M. Kebede N. Wang H. V. Showrin S. . (2026). Temporal regulation of human reactive astrocytes reveals their capacity for antigen presentation. Neuron 114, 2331–2346. doi: 10.1016/j.neuron.2026.02.025 41916282 PMC13060027

[B19] Hill R. A. Li A. M. Grutzendler J. (2018). Lifelong cortical myelin plasticity and age-related degeneration in the live mammalian brain. Nat. Neurosci. 21, 683–695. doi: 10.1038/s41593-018-0120-6 29556031 PMC5920745

[B20] Hughes E. G. Kang S. H. Fukaya M. Bergles D. E. (2013). Oligodendrocyte progenitors balance growth with self-repulsion to achieve homeostasis in the adult brain. Nat. Neurosci. 16, 668–676. doi: 10.1038/nn.3390 23624515 PMC3807738

[B21] Hughes E. G. Orthmann-Murphy J. L. Langseth A. J. Bergles D. E. (2018). Myelin remodeling through experience-dependent oligodendrogenesis in the adult somatosensory cortex. Nat. Neurosci. 21, 696–706. doi: 10.1038/s41593-018-0121-5 29556025 PMC5920726

[B22] Ioannou M. S. Jackson J. Sheu S. H. Chang C. L. Weigel A. V. Liu H. . (2019). Neuron-astrocyte metabolic coupling protects against activity-induced fatty acid toxicity. Cell 177, 1522–1535. doi: 10.1016/j.cell.2019.04.001 31130380

[B23] Jakel S. Agirre E. Mendanha Falcao A. Van Bruggen D. Lee K. W. Knuesel I. . (2019). Altered human oligodendrocyte heterogeneity in multiple sclerosis. Nature. 566, 543–547. doi: 10.1038/s41586-019-0903-2 30747918 PMC6544546

[B24] Kierdorf K. Masuda T. Jordao M. J. C. Prinz M. (2019). Macrophages at CNS interfaces: ontogeny and function in health and disease. Nat. Rev. Neurosci. 20, 547–562. doi: 10.1038/s41583-019-0201-x 31358892

[B25] Kirby L. Jin J. Cardona J. G. Smith M. D. Martin K. A. Wang J. . (2019). Oligodendrocyte precursor cells present antigen and are cytotoxic targets in inflammatory demyelination. Nat. Commun. 10:3887. doi: 10.1038/s41467-019-11638-3 31467299 PMC6715717

[B26] Lee J. H. Kim J. Y. Noh S. Lee H. Lee S. Y. Mun J. Y. . (2021). Astrocytes phagocytose adult hippocampal synapses for circuit homeostasis. Nature 590, 612–617. doi: 10.1038/s41586-020-03060-3 33361813

[B27] Liu Y. U. Ying Y. Li Y. Eyo U. B. Chen T. Zheng J. . (2019). Neuronal network activity controls microglial process surveillance in awake mice via norepinephrine signaling. Nat. Neurosci. 22, 1771–1781. doi: 10.1038/s41593-019-0511-3 31636449 PMC6858573

[B28] McKenzie I. A. Ohayon D. Li H. De Faria J. P. Emery B. Tohyama K. . (2014). Motor skill learning requires active central myelination. Science 346, 318–322. doi: 10.1126/science.1254960 25324381 PMC6324726

[B29] Micu I. Plemel J. R. Caprariello A. V. Nave K. A. Stys P. K. (2018). Axo-myelinic neurotransmission: a novel mode of cell signalling in the central nervous system. Nat. Rev. Neurosci. 19, 49–58. doi: 10.1038/nrn.2017.128 29118449

[B30] Mrdjen D. Pavlovic A. Hartmann F. J. Schreiner B. Utz S. G. Leung B. P. . (2018). High-dimensional single-cell mapping of central nervous system immune cells reveals distinct myeloid subsets in health, aging, and disease. Immunity 48, 380–395. doi: 10.1016/j.immuni.2018.01.011 29426702

[B31] Murphy-Royal C. Ching S. Papouin T. (2023). A conceptual framework for astrocyte function. Nat. Neurosci. 26, 1848–1856. doi: 10.1038/s41593-023-01448-8 37857773 PMC10990637

[B32] Nave K. A. Asadollahi E. Sasmita A. (2023). Expanding the function of oligodendrocytes to brain energy metabolism. Curr. Opin. Neurobiol. 83:102782. doi: 10.1016/j.conb.2023.102782 37703600

[B33] Pan S. Mayoral S. R. Choi H. S. Chan J. R. Kheirbek M. A. (2020). Preservation of a remote fear memory requires new myelin formation. Nat. Neurosci. 23, 487–499. doi: 10.1038/s41593-019-0582-1 32042175 PMC7213814

[B34] Pandey S. Shen K. Lee S. H. Shen Y. A. Wang Y. Otero-Garcia M. . (2022). Disease-associated oligodendrocyte responses across neurodegenerative diseases. Cell Rep. 40:111189. doi: 10.1016/j.celrep.2022.111189 36001972

[B35] Paolicelli R. Sierra A. Stevens B. Tremblay M.-E. Aguzzi A. Ajami B. . (2022). Defining microglial states and nomenclature: a roadmap to 2030. Cell. 110, 3458–3483. doi: 10.1016/j.neuron.2022.10.020 36327895 PMC9999291

[B36] Patani R. Hardingham G. E. Liddelow S. A. (2023). Functional roles of reactive astrocytes in neuroinflammation and neurodegeneration. Nat. Rev. Neurol. 19, 395–409. doi: 10.1038/s41582-023-00822-1 37308616

[B37] Pereira-Iglesias M. Maldonado-Teixido J. Melero A. Piriz J. Galea E. Ransohoff R. M. . (2025). Microglia as hunters or gatherers of brain synapses. Nat. Neurosci. 28, 15–23. doi: 10.1038/s41593-024-01818-w 39663381

[B38] Rustenhoven J. Kipnis J. (2022). Brain borders at the central stage of neuroimmunology. Nature 612, 417–429. doi: 10.1038/s41586-022-05474-7 36517712 PMC10205171

[B39] Steadman P. E. Xia F. Ahmed M. Mocle A. J. Penning A. R. A. Geraghty A. C. . (2020). Disruption of oligodendrogenesis impairs memory consolidation in adult mice. Neuron 105, 150–164. doi: 10.1016/j.neuron.2019.10.013 31753579 PMC7579726

[B40] Tansley S. Gu N. Guzman A. U. Cai W. Wong C. Lister K. C. . (2022). Microglia-mediated degradation of perineuronal nets promotes pain. Science 377, 80–86. doi: 10.1126/science.abl6773 35617374

[B41] Tewari B. P. Chaunsali L. Prim C. E. Sontheimer H. (2022). A glial perspective on the extracellular matrix and perineuronal net remodeling in the central nervous system. Front. Cell. Neurosci. 16:1022754. doi: 10.3389/fncel.2022.1022754 36339816 PMC9630365

[B42] Tewari B. P. Woo A. M. Prim C. E. Chaunsali L. Patel D. C. Kimbrough I. F. . (2024). Astrocytes require perineuronal nets to maintain synaptic homeostasis in mice. Nat. Neurosci. 27, 1475–1488. doi: 10.1038/s41593-024-01714-3 39020018 PMC11303255

[B43] Umpierre A. D. Bystrom L. L. Ying Y. Liu Y. U. Worrell G. Wu L. J. (2020). Microglial calcium signaling is attuned to neuronal activity in awake mice. Elife 9:e56502. doi: 10.7554/eLife.56502.sa2 32716294 PMC7402678

[B44] Van Der Vliet D. Di X. Shamorkina T. M. Coulon-Bainier C. Pavlovic A. Van Der Vliet I. . (2026). Foamy microglia link oxylipins to disease progression in multiple sclerosis. Nat. Neurosci. 29, 1585–1598. doi: 10.1038/s41593-026-02302-3 42168651 PMC13337515

[B45] Xin W. Chan J. R. (2020). Myelin plasticity: sculpting circuits in learning and memory. Nat. Rev. Neurosci. 21, 682–694. doi: 10.1038/s41583-020-00379-8 33046886 PMC8018611

[B46] Zia S. Hammond B. P. Zirngibl M. Sizov A. Baaklini C. S. Panda S. P. . (2022). Single-cell microglial transcriptomics during demyelination defines a microglial state required for lytic carcass clearance. Mol. Neurodegener. 17:82. doi: 10.1186/s13024-022-00584-2 36514132 PMC9746011

[B47] Zia S. Rawji K. S. Michaels N. J. Burr M. Kerr B. J. Healy L. M. . (2020). Microglia diversity in health and multiple sclerosis. Front. Immunol. 11:588021. doi: 10.3389/fimmu.2020.588021 33240276 PMC7677361

[B48] Ziaee S. M. Nemati S. Kataria H. Jakova E. Hosseini S. M. Ghelich P. . (2026). Neuregulin-1 facilitates myelin regeneration through microglia-mediated mechanisms in a mouse model of chronic demyelination. Nat. Commun. 17, 1–30. doi: 10.1038/s41467-026-72639-7 42115623 PMC13309555

